# Reproducibility of Manual Periodontal Probing Following a Comprehensive Standardization and Calibration Training Program

**DOI:** 10.13188/2377-987X.1000063

**Published:** 2022-06-29

**Authors:** Bryan P. Fitzgerald, Charles E. Hawley, Charles Q. Harrold, J. Steven Garrett, Alan M. Polson, Thomas E. Rams

**Affiliations:** 1 Formerly Division of Periodontics, University of Maryland School of Dentistry, Baltimore, Maryland, USA; 2 Department of Periodontology, Tufts University School of Dental Medicine, Boston, Massachusetts, USA; 3 Formerly Department of Surgical Sciences, University of Colorado School of Dental Medicine, Aurora, Colorado; presently retired, Chandler, Arizona, USA; 4 Formerly Atrix Laboratories, Inc., Ft. Collins, Colorado, USA; presently retired, Rigby, Idaho, USA; 5 Department of Periodontics, University of Pennsylvania School of Dental Medicine, Philadelphia, Pennsylvania, USA; 6 Department of Periodontology and Oral Implantology, Temple University School of Dentistry, Philadelphia, Pennsylvania, USA

**Keywords:** Diagnosis, Periodontal diseases, Periodontal pocket, Periodontal attachment loss, Gingival bleeding on probing

## Abstract

**Background:**

Clinical standardization and calibration training is recommended to increase the reproducibility of periodontal probing, but its impact on manual periodontal probing outcomes has received little attention. This study examined the reproducibility of manual periodontal probing performed by a periodontist after completion of a comprehensive standardization and calibration training program.

**Methods:**

A newly-educated periodontist was subjected to an individualized periodontal probing standardization and calibration training program involving approximately 24 total hours of lecture, bench-top, and clinical instruction/evaluation. Satisfactory completion of each portion of the training program required ≥ 95% intra-examiner agreement within 1 mm between initial and repeat measurements, and a ≥ 90% level of exact agreement with measurements by a “gold standard” examiner. The periodontist then evaluated bleeding on probing (BOP) and performed duplicate measurements of probing depth (PD) and the distance between the cementoenamel junction and gingival margin (CEJ-GM) with a manual periodontal probe on 567 periodontal sites exhibiting ≥ 5 mm PD with BOP in 39 adults. Clinical periodontal attachment level (CAL) was calculated for each site as (PD) - (CEJ-GM).

**Results:**

Intra-examiner measurement error (the standard deviation for a single measurement) was found to be 0.21 mm for PD, 0.15 mm for CEJ-GM, and 0.26 mm for CAL. Replicate assessments of PD and CAL yielded excellent exact agreement kappa scores of 0.86 and 0.87, respectively. Greater intra-examiner measurement error was found at periodontal sites with more gingival inflammation as measured by higher BOP index scores.

**Conclusion:**

These findings demonstrate that a rigorous periodontal probing standardization and calibration training program facilitates acquisition of highly reproducible PD and CAL assessments in moderate to deep inflamed periodontal pockets with a manual periodontal probe. Similar formal hands-on training should be incorporated into dental education programs and clinical research studies to improve the diagnostic performance of manual periodontal probing of the periodontium.

## Introduction

Periodontal probing provides the foundation for clinical examination of the periodontium, helping determine periodontal diagnosis and evaluate therapeutic outcomes in patients [1,2]. However, accurate and reproducible physical probing of the periodontium is clinically challenging to carry out, increasing the likelihood of probing measurement errors. Variability in clinical examiner experience, probing force, degree of gingival tissue inflammation, tooth root anatomy, probing depth, periodontal site location, probe subgingival insertion angles, probe tip diameter, probe millimeter markings, visual reading of probe markings, rounding off of probe measurements, patient cooperation, extent of subgingival calculus, and transcription recording errors, may contribute to periodontal probe measurement errors [1,3,4].

Diagnosis of periodontitis is dependent upon reliable periodontal probing. Clinical periodontal attachment level (CAL) provides a clinical approximation of connective tissue attachment to tooth root surfaces [1]. Manual probing can identify the coronal extent of CAL to within ≤ 0.55 mm of histologic findings [5], but underestimates the total root surface area affected by CAL loss [6]. CAL may be directly measured with a periodontal probe from the cementoenamel junction of teeth [7], but is more frequently calculated from separate measurements of probing depth (PD) and the distance between the cementoenamel junction and gingival margin (CEJ-GM), with CAL = (PD) - (CEJ-GM) [3].

CAL measurements are widely recognized as the “gold standard” for identifying progressive periodontitis in patients [1]. Lindhe et al. designated a ≥ 3 mm change in CAL to detect progressive periodontitis sites in untreated patients [8]. This was based on the view that CAL changes exceeding three times the standard deviation (SD) of replicate CAL measurements with a manual periodontal probe, which were previously reported to be 0.82 mm on severe periodontitis patients [9], were unlikely to be due to examiner measurement error. Improved manual probe reproducibility may enable use of a lower CAL change threshold for detecting progressive periodontitis and increase the diagnostic sensitivity of manual periodontal probes.

To increase the reproducibility of periodontal probing, formal standardization and calibration training [10], also known as examiner alignment and assessment training [11], is recommended to identify and minimize sources of clinical examiner variation in probing assessments. Abbas et al. reported improved reproducibility in PD assessments after clinicians viewed a video program on standardization of periodontal probing procedures [12].

However, the effectiveness of more extensive and rigorous hands-on training programs is not known. To address this issue, the present study examined the reproducibility of periodontal probing measurements attained by a newly-educated periodontist following the completion of a comprehensive periodontal probing standardization and calibration training program.

## Materials & Methods

This study involved a secondary retrospective analysis of periodontal probing reproducibility data from one study site (University of Maryland School of Dentistry, Baltimore, Maryland, USA) participating in a previous US Food and Drug Administration (FDA)-approved phase-3 product evaluation of 10% doxycycline hyclate in a biodegradable drug delivery system [13]. The reproducibility data were obtained after the study patients provided signed informed consent, consistent with the Helsinki Declaration of 1975, as revised in 2013, and as approved by the human subjects institutional review board at the University of Maryland at Baltimore. The present data analysis was also approved by the Temple University human subjects institutional review board.

### Pre-Study Examiner Standardization and Calibration Training

A newly-educated periodontist with no research experience (author BPF) was the single periodontist examiner for the periodontal probing reproducibility study. Because of his relative clinical periodontal inexperience, no baseline reproducibility assessments of his periodontal probing technique were made. Prior to the start of the probing reproducibility study, he underwent individualized pre-study periodontal probing standardization and calibration training involving approximately 24 total hours of lecture, bench-top exercises, clinical instruction, and evaluation [10,14]. An initial half-day didactic review of periodontal data collection principles and procedures focused on use of consistent manual probing forces, identification of interproximal tooth contact points, proper periodontal probe alignment, rounding-up or down rules, appropriate reference points, identification of CEJ and GM location, PD measurement, CAL calculation, and scoring of BOP. Following this, laboratory bench-top probing exercises with dentiform models depicting various types of periodontitis lesions were completed under supervision of two “gold standard” experienced periodontists previously documented to possess a high level of inter-examiner reliability with each other (authors CQH and AMP). Full-mouth clinical inter- and intra-examiner probing exercises were then conducted on four pre-study periodontitis patients with the “gold standard” examiners at a location extramural (University of Colorado School of Dental Medicine, Aurora, Colorado, USA) to the probing reproducibility study site in Baltimore. A standard UNC-15 probe (UNC #15, Hu-Friedy, Chicago, Illinois, USA) was used throughout the calibration exercises. Subsequently, five additional pre-study periodontitis patients at the probing reproducibility study site were subjected to supervised full-mouth replicate periodontal probings, with a final inter- and intra-examiner probing calibration carried out on two more pre-study periodontitis patients at the reproducibility study site with a “gold standard” examiner (author CQH). All of the 11 pre-study periodontitis patients exhibited a similar range of PD, CEJ-GM distance, and BOP as patients in the subsequent probing reproducibility study. A ≥ 95% intra-examiner agreement within 1 mm between initial and repeat measurements, and a ≥ 90% level of exact agreement with “gold standard” examiner measurements, was required for satisfactory completion of each portion of the pre-study examiner standardization and calibration training program.

### Patients

After completion of the pre-study standardization and calibration training program, the periodontist examiner conducted periodontal probing reproducibility examinations on 39 systemically-healthy adults (20 male, 19 female; aged 32–65 years; mean age 47.8 ± 8.1 (SD) years), who presented with localized to generalized severe periodontitis (equivalent to Stage III/Grade B periodontitis) [2], where at least two dentition quadrants had at least four periodontal sites exhibiting ≥ 5 mm PD with BOP, of which at least two of these sites had PD ≥ 7 mm.

Patients were excluded if they had been treated with periodontal root scaling within the prior two months.

### Clinical Measurements

**Figure 1:**
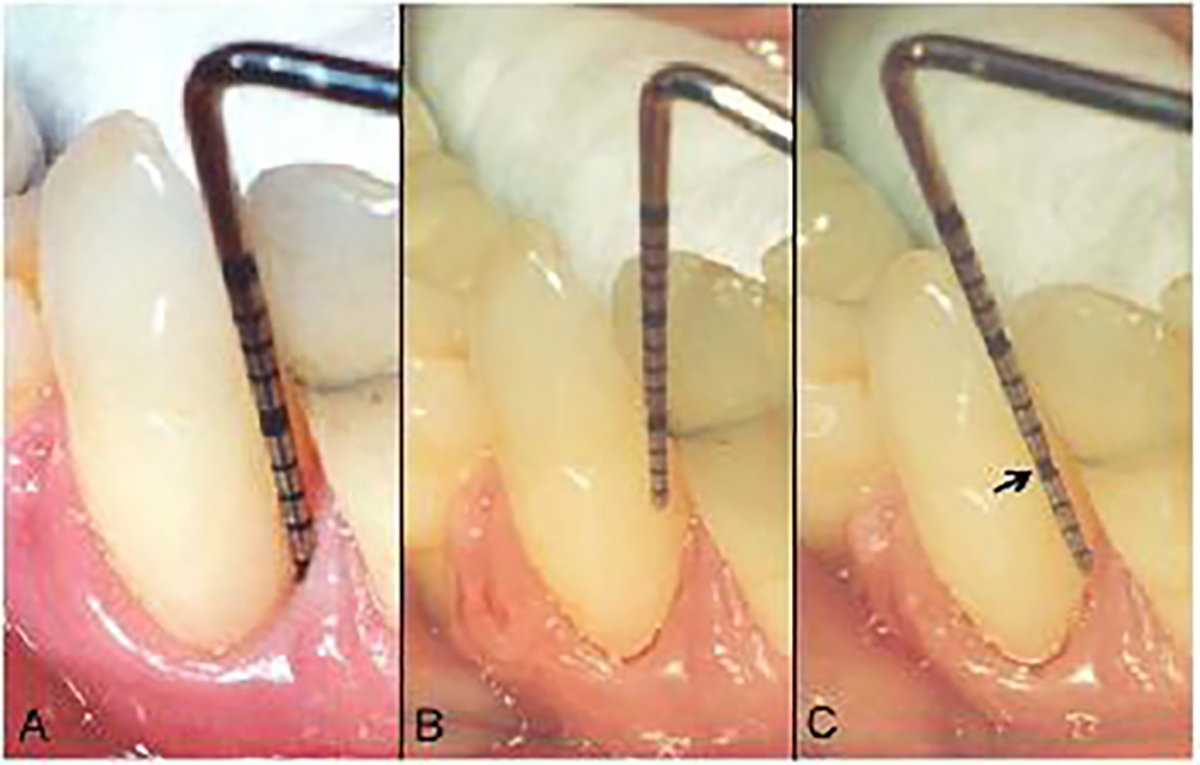
Probing depth measurement (A), Detection of supragingival cementoenamel junction with periodontal probe tip at 45° angle to long axis of tooth surface (B), and measurement of distance between cementoenamel junction (arrow) and gingival margin (C).

Clinical measurements were assessed at six sites per tooth in each study patient. PD was measured to the nearest whole millimeter from the gingival margin to the most apical gingival tissue penetration of the probe tip using a UNC-15 periodontal probe, with the probe inserted with its long axis aligned parallel to the long axis of the tooth (Figure 1A). Interproximal PD measurements were carried out immediately adjacent to interproximal tooth contact points. If there was no interproximal contact present, the periodontal sites were excluded from analysis.

BOP was scored on a 0–3 index scale after measurement of PD. Bleeding at each periodontal site was graded as follows: 0 = no bleeding; 1 = delayed single bleeding point, or a fine line of blood (Figure 1B); 2 = interdental triangle becomes filled; 3 = immediate profuse bleeding after probing [15].

The CEJ-GM distance was then determined by initially placing the periodontal probe tip against the enamel surface coronal to the margin of the gingiva at a 45° angle to the long axis of the tooth (Figure 1B) [3]. When the CEJ was located subgingival to the gingival margin, the probe tip was moved apically with minimal force into the gingival sulcus while maintaining contact with the tooth surface. The CEJ location was then detected by tactile sensation or by observation of a change in the direction of the periodontal probe tip movement during advancement from the tooth enamel to cementum. The probe tip was moved in a coronal direction from the gingival margin if the CEJ was located coronal to the gingival margin and difficult to visually discern. The CEJ-GM distance was measured to the nearest whole millimeter, with positive numbers recorded if the most coronal aspect of the gingival tissue margin was located on enamel, and negative values recorded when the most coronal aspect of the gingival tissue margin was located apical to the CEJ on cementum (Figure 1C). Only periodontal sites where the CEJ could be clinically located were included in the present analysis, with exclusion of sites where the CEJ was obscured by margins of dental restorations. CAL was calculated from measurements of PD and CEJ-GM, with CAL = (PD) - (CEJ-GM) [3].

### Examination Procedures

**Figure 2:**
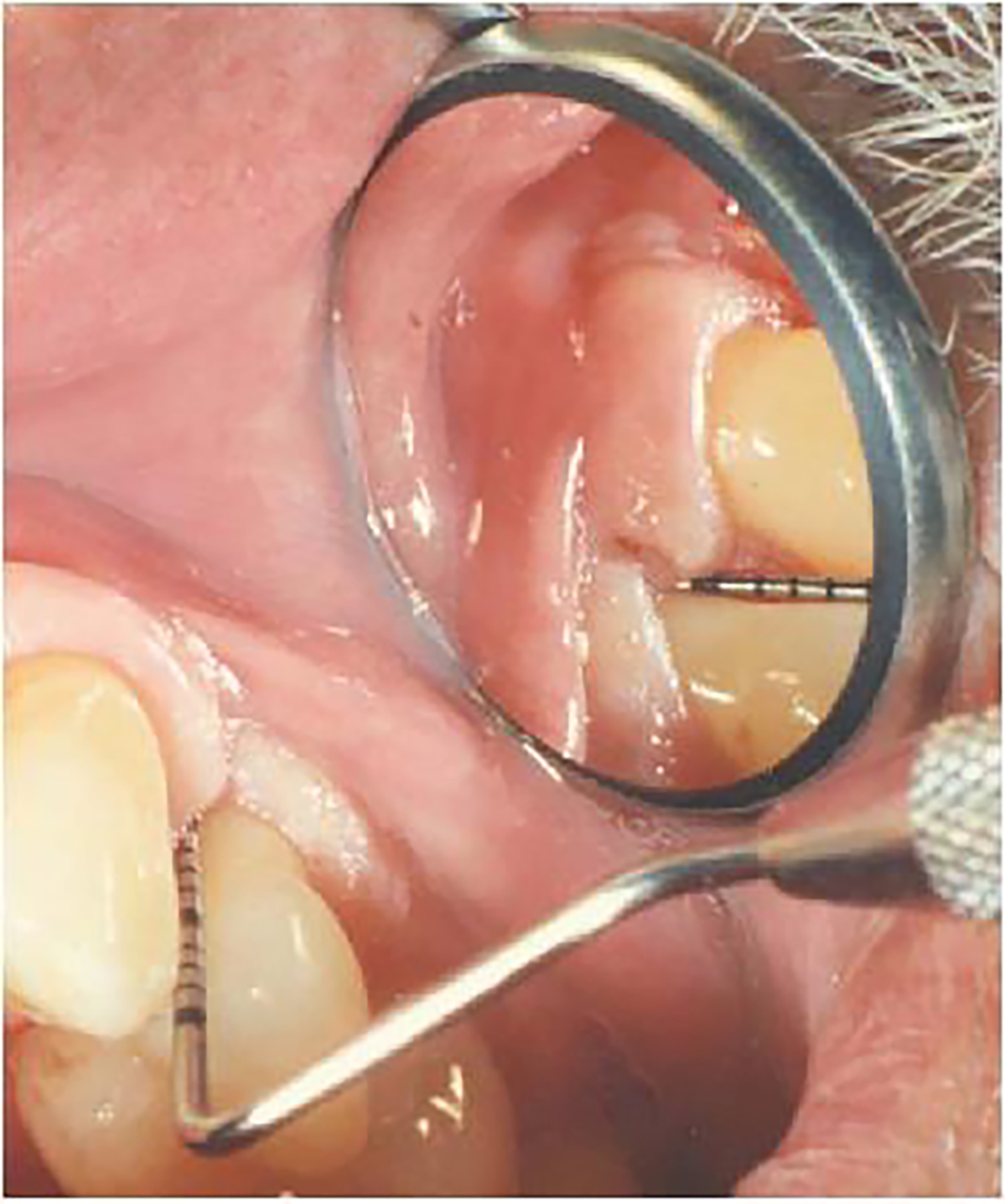
Use of mirror-assisted indirect vision for assessing periodontal probe measurements on mesial-buccal surface of maxillary first premolar.

The clinical examinations were conducted by the trained and calibrated periodontist examiner. Cotton roll isolation and air drying were used to establish a dry field during the clinical examination procedures, with measurement values verbally called out to a data recording assistant for transcription. In order to standardize data collection procedures at all periodontal sites irrespective of their intraoral location, as required by the FDA-approved product evaluation protocol, all clinical measurements at both facial and lingual tooth surfaces were obtained using mirror-assisted indirect vision (Figure 2). The clinical examination sequence on each study patient started with a whole-mouth assessment of the Plaque Index on all periodontal sites [16]. Then, using a UNC-15 probe, PD measurements and scoring of the BOP index were carried out on the maxillary right dentition quadrant. These values were evaluated by the study site principal investigator (author CEH), independent of the periodontist examiner, to identify periodontal sites exhibiting a combination of PD ≥ 5 mm and BOP for probing reproducibility assessments. The CEJ-GM distance was then measured on these designated periodontal sites throughout the dentition quadrant. These same steps were then carried out in turn on the maxillary left, mandibular left, and mandibular right dentition quadrants. After all initial data collection was completed in all dentition quadrants, repeat measurements of PD and CEJ-GM on the designated periodontal sites in each dentition quadrant were performed, as specified by the study center principal investigator through the data recording assistant. Repeat assessments were initiated first in the maxillary right dentition quadrant, followed in turn by the maxillary left, mandibular left, and mandibular right dentition quadrants. Repeat measurements were obtained at least 15 minutes after initial evaluations to reduce the effect of examiner memory of initial recordings [10]. The periodontist examiner was kept blinded to initial measurement values.

### Data Analysis

Data analysis was performed using a statistical computer software package (Statistical Analysis System, SAS Institute, Inc., Cary, North Carolina, USA). Mean values and the SD of differences between initial and replicate periodontal site assessments were calculated for PD, CEJ-GM distance, and CAL. Intra-examiner measurement error (ME) for each of the probing assessments was estimated by calculating the SD for a single measurement as follows [17]:

$$(ME)={\hat{\sigma }}_{ij}=\sqrt{\frac{1}{2\sum_{i=1}^{n}n_{i}}\sum_{i=1}^{n}\sum_{j=1}^{n_{i}}{\left(y_{ij1}-y_{ij2}\right)}^{2}}=\sqrt{\frac{1}{2\sum_{i=1}^{n}n_{i}}\sum_{i=1}^{n}\sum_{j=1}^{n_{i}}d_{ij}^{2}}$$

Where each σ_ij_ at a site level is estimable based on the difference between replicate measurements d_ij_ (representing the difference in replicate scores for the observed measurement, y_ij_, for the jth site within the ith patient; n = number of patients), and where one assumes measurement errors are independent of patient and site-type, i.e., var (y_ijk_) = σ^2^.

Kappa statistics were used to quantify intra-examiner agreement beyond chance for site-based replicate assessments of PD and CAL [18]. Exact kappa and a kappa value combining pairs of scores within 1 mm of each other were calculated for replicate assessments of PD and CAL, as previously described [19,20], but without confidence intervals adjusted for within-patient effects. Kappa values between 0.40 and 0.75 were considered to represent fair to good agreement, with kappa > 0.75 indicating excellent agreement [18].

The influence of gingival inflammation on intra-examiner reproducibility of PD, CEJ- GM distance, and CAL was evaluated by comparing their reproducibility parameters across increasing BOP index scores.

Patients in the lowest and highest quintile of patients ranked by the total number of periodontal sites/patient subjected to replicate evaluations were also compared relative to agreement attained within 1 mm for replicate PD, CEJ-GM distance, and CAL measurements.

## Results

A total of 567 periodontal pockets demonstrating ≥ 5 mm PD and BOP were evaluated in the probing reproducibility examinations, of which 468 (82.5%) were located on interproximal tooth surfaces, with each of the 39 study patients contributing 7–20 periodontal sites from at least two dentition quadrants (Table 1). Among other sites in the study patients not exhibiting ≥ 5 mm PD and BOP, 0.2% were ≥ 5 mm PD but without BOP, 70.7% were < 5 mm with BOP, and 29.1% were < 5 mm but without BOP.

Table 2 presents the mean initial and replicate measurements for PD, CEJ-GM distance, and CAL for the 567 periodontal pockets. PD values averaged 5.7 mm (range 5–14 mm), with mean differences among various tooth sites found to be ≤ 0.10 mm between replicate values for PD, CEJ-GM distance, and CAL. The SD for single measurements (intra-examiner measurement error) of PD, CEJ-GM distance, and CAL was 0.21 mm, 0.15 mm and 0.26 mm, respectively (Table 2).

Table 3 reveals that exact agreement was found for 92.1% of replicate PD measurements, and for 89.1% of replicate CAL assessments, with both parameters attaining agreement within 1 mm for more than 99% of examined periodontal sites. Kappa values for exact intra-examiner agreement were 0.86 for PD (0.99 for agreement within 1 mm), and 0.87 for CAL (0.98 for agreement within 1 mm). These kappa values all exceeded the threshold required (kappa > 0.75) to indicate excellent intra-examiner agreement [18].

Among specific tooth surfaces, lingual surfaces generally yielded lower intra-examiner measurement error values and higher kappa scores, as compared to buccal tooth surfaces for both PD and CAL (Tables 2 and 3).

Table 4 shows the influence of BOP index scores on the reproducibility of PD, CEJ-GM distance, and CAL assessments. Higher BOP index scores were associated with greater intra-examiner measurement error for all of the probing evaluations carried out, and decreased kappa values for CAL (Table 4).

All PD and CEJ-GM replicate measurements, and all but two CAL replicate measurements (one in each patient group), were within 1 mm of each other in patients where only a few periodontal sites were subjected to replicate evaluations (lowest quintile; 7–12 sites per patient; 89 total sites in 8 patients), as well as in patients where a higher number of periodontal sites were scored twice (highest quintile; 18–20 sites per patient; 155 total sites in 8 patients).

## Discussion

The present study findings demonstrate that a manual periodontal probe visually read to the nearest whole millimeter may provide highly reproducible PD and CAL measurements on moderate to deep inflamed periodontal pockets when employed by a rigorously trained and calibrated periodontist. These findings agree with and extend previous studies of manual probes [9,20–24], where most replicate CAL measurements were made on periodontal sites with shallow to moderate probing depths, and variable levels of gingival health and inflammation. In contrast, the present study evaluated only moderate to deep (5–14 mm) periodontal pockets with BOP, which are clinically more challenging to physically probe and subject to greater periodontal probe variation.

A remarkably low SD for a single CAL assessment of 0.26 mm (intra-examiner error) was attained in the present study. This value is strikingly better than the SD of single CAL assessments, as calculated by Yang et al. [25], that range from 0.54 mm to 0.69 mm for manual periodontal probes [9,22,26,27], and similar to values of 0.20 mm to 0.31 mm found for a controlled-force probe used with an acrylic occlusal reference stent [25,28]. However, a controlled-force periodontal probe with automated CEJ detection was reported to provide a lower intra-examiner error of approximately 0.12 mm [29].

Several reasons may account for the markedly better CAL reproducibility achieved in the present study as compared to previous investigations of manual periodontal probes [9,20–24,26,27]. Only a single periodontist performed replicate CAL assessments in the present study, in contrast to Haffajee et al. where three different examiners were used [9]. Evaluations in the present study were under ideal clinical examination conditions, as compared to a replication study conducted outdoors with portable dental chairs and no compressed air or suction [20]. An extensive and well-defined pre-study standardization and calibration training program was completed by the periodontist in the present study, whereas most previous studies did not conduct or failed to report details of any examiner training and calibration exercises. The periodontist in the present study may also have been particularly gifted with regard to the patience and temperament needed to carry out accurate and reproducible replicate probing, and an ability to apply a uniform probing force at standardized probe insertion angles, properly identify the CEJ on subgingival tooth surfaces, and accurately read probe markings.

It is additionally possible that the periodontist recalled initial measurement values when performing replicate evaluations, particularly in patients where a small number of periodontal sites were examined twice. However, the standardized examination protocol, where assessments of other parts of the dentition and at least 15 minutes transpired between replicate site measurements, helped mitigate against this possibility. The high probe reproducibility found in patients with many sites scored twice (highest quintile of sites/patient; where examiner recall is less likely), was similar to patients where replicate evaluations were performed on only a few sites (lowest quintile of sites/patient; where examiner recall is more likely), suggesting that examiner performance in the present study was not predominately due to recall bias. However, it remains to be established if other dental professionals can attain and maintain over time similar levels of probing reproducibility when exposed to the standardization and calibration training program employed in the present study. If so, then longitudinal monitoring of CAL in clinical practice and periodontal research studies may be reliably performed with a manual probe, instead of a controlled-force probe with an acrylic occlusal reference stent, since comparable levels of intra-examiner error are found between them (0.26 mm with manual probe in this study versus 0.20–0.31 mm previously reported with controlled-force probe [25,28]). Better periodontal probe reproducibility may also help reduce sample sizes needed in clinical research studies to identify statistically significant differences between outcome variables scored with a manual probe [30].

Importantly, the excellent probe reproducibility attained by the periodontist in the study patients corresponded as well or better with reproducibility levels achieved in the pre-study standardization and calibration training program. This supports the concept that examiner performance attained during standardization and calibration training is a critical determinant of a clinician’s subsequent reliability in making accurate and consistent periodontal measurements in clinical practice settings and research studies.

Of the clinical components used to calculate CAL, there was less intra-examiner error for a single measurement of the CEJ-GM distance (0.15 mm) than for PD (0.21 mm), even though the CEJ may be difficult to locate in subgingival and interproximal locations [1,3,26]. However, CEJ-GM distance measurements are generally smaller values possessing less potential variability than usually larger PD measurements, and are not influenced by gingival inflammation in adjacent soft tissues. In contrast, PD values show more variability as a result of varying periodontal probe tip penetration into inflamed gingival connective tissues subjacent to the junctional epithelium [31,32]. The present study findings that PD and CAL measurements on moderate to deep periodontal pockets are less reproducible with higher BOP index scores (reflecting greater levels of gingival inflammation) are consistent with these histologic observations [31,32].

The increased degree of reproducibility in PD and CAL measurements on lingual as compared to buccal tooth surfaces in the present study (Tables 2 and 3) is likely related to unique methodological procedures required by the FDA-sanctioned phase-3 clinical product evaluation protocol, where measurements were made using mirror-assisted indirect vision on both buccal and lingual tooth surfaces, an approach not otherwise employed in clinical practice. This unusual clinical examination approach posed technical performance difficulties for the periodontist examiner on buccal tooth surfaces, particularly mesio-buccal sites. In comparison, greater reproducibility in PD and CAL measurements is reported for buccal tooth sites in previous studies [20,22,23], where examiners employed direct visualization for buccal tooth surfaces and mirror-assisted indirect vision for lingual surfaces, which likely enhanced and hindered probe readings, respectively.

## Conclusion

A newly-educated periodontist, after completing a rigorous periodontal probing standardization and calibration training program, was able to obtain highly reproducible PD and CAL assessments in moderate to deep inflamed periodontal pockets using a manual periodontal probe. Similar formal hands-on training should be incorporated to a greater extent into dental education programs and clinical research studies to improve the diagnostic performance of manual periodontal probing of the periodontium.

## Figures and Tables

**Table 1:** Distribution by tooth surface of inflamed periodontal pockets with ≥ 5 mm PD.

Tooth surface	No. (%) of sites
mesio-buccal	127 (22.4)
mid-buccal	36 (6.3)
disto-buccal	82 (14.5)
mesio-lingual	179 (31.6)
mid-lingual	63 (11.1)
disto-lingual	80 (14.1)
All sites	567 (100)

**Table 2:** Mean replicate probing measurements (mm ± SD) and measurement errors (mm) by tooth surface for inflamed periodontal pockets with ≥ 5 mm PD.

Probing assessment and tooth surface	Initial measurement	Replicate measurement	Difference between initial and replicate measurements	Measurement error (SD for single measurement)
**PD**				
mesio-buccal	5.59 ± 0.91	5.54 ± 0.88	0.05 ± 0.35	0.25
mid-buccal	5.67 ± 0.89	5.72 ± 0.85	−0.05 ± 0.33	0.24
disto-buccal	5.68 ± 1.14	5.70 ± 1.16	−0.02 ± 0.35	0.25
mesio-lingual	5.72 ± 1.12	5.77 ± 1.18	−0.05 ± 0.30	0.21
mid-lingual	5.73 ± 1.02	5.76 ± 1.04	−0.03 ± 0.18	0.13
disto-lingual	5.50 ± 0.84	5.46 ± 0.84	0.04 ± 0.19	0.14
All surfaces	5.65 ± 1.02	5.66 ± 1.04	−0.01 ± 0.30	0.21
**CEJ-GM distance**				
mesio-buccal	1.26 ± 1.59	1.24 ± 1.58	0.02 ± 0.15	0.11
mid-buccal	0.11 ± 1.60	0.11 ± 1.60	0.00 ± 0.00	0.00
disto-buccal	0.79 ± 1.82	0.80 ± 1.84	−0.01 ± 0.11	0.08
mesio-lingual	1.54 ± 1.50	1.48 ± 1.50	0.06 ± 0.24	0.18
mid-lingual	0.47 ± 1.70	0.44 ± 1.70	0.03 ± 0.25	0.18
disto-lingual	1.60 ± 1.70	1.54 ± 1.66	0.06 ± 0.24	0.18
All surfaces	1.17 ± 1.69	1.13 ± 1.68	0.04 ± 0.20	0.15
**CAL**				
mesio-buccal	6.86 ± 1.83	6.79 ±1.87	0.07 ± 0.40	0.29
mid-buccal	5.77 ± 1.91	5.83 ± 1.88	−0.06 ± 0.33	0.24
disto-buccal	6.47 ± 2.24	6.51 ± 2.32	−0.04 ± 0.37	0.26
mesio-lingual	7.25 ± 1.81	7.24 ± 1.80	0.01 ± 0.39	0.28
mid-lingual	6.21 ± 1.91	6.21 ± 1.95	0.00 ± 0.31	0.22
disto-lingual	7.10 ± 1.93	7.00 ± 1.89	0.10 ± 0.30	0.22
All surfaces	6.82 ± 1.96	6.80 ± 1.97	0.02 ± 0.37	0.26

**Table 3:** Reproducibility of PD and CAL by tooth surface for inflamed periodontal pockets with ≥ 5 mm PD.

Probing assessment and tooth surface	% exact agreement / % agreement within 1 mm	Exact kappa / kappa within 1 mm
**PD**		
mesio-buccal	89.8 / 99.2	0.81 / 0.96
mid-buccal	88.9 / 100	0.82 / 1.00
disto-buccal	87.8 / 100	0.79 / 1.00
mesio-lingual	92.7 / 99.4	0.88 / 0.98
mid-lingual	96.8 / 100	0.95 / 1.00
disto-lingual	96.3 / 100	0.92 / 1.00
All surfaces	92.1 / 99.6	0.86 / 0.99
**CAL**		
mesio-buccal	88.2 / 98.4	0.86 / 0.97
mid-buccal	88.9 / 100	0.87 / 1.00
disto-buccal	86.6 / 100	0.84 / 1.00
mesio-lingual	88.3 / 98.9	0.86 / 0.98
mid-lingual	95.2 / 98.4	0.94 / 0.97
disto-lingual	90.0 / 100	0.88 / 1.00
All surfaces	89.1 / 99.1	0.87 / 0.98

**Table 4:** Reproducibility of probing assessments by BOP index scores for inflamed periodontal pockets with ≥ 5 mm PD.

Probing assessment and BOP index score	SD of differences in replicate measurements, mm	Measurement error (SD for single measurement, mm)	% exact agreement	Exact kappa
**PD**				
BOP index = 1	0.24	0.17	94.3	0.84
BOP index = 2	0.30	0.21	92.0	0.84
BOP index = 3	0.31	0.22	91.6	0.88
CEJ-GM distance				
BOP index = 1	0.00	0.00	ND	ND
BOP index = 2	0.20	0.14	ND	ND
BOP index = 3	0.23	0.17	ND	ND
CAL				
BOP index = 1	0.17	0.17	94.3	0.93
BOP index = 2	0.37	0.26	89.1	0.87
BOP index = 3	0.39	0.28	87.6	0.85

ND = not determined
